# Characterization and performance evaluation of Cu-based/TiO_2_ nano composites

**DOI:** 10.1038/s41598-022-10616-y

**Published:** 2022-04-23

**Authors:** D. Saber, Kh. Abd El-Aziz, Bassem F. Felemban, Abdulaziz H. Alghtani, Hafiz T. Ali, Emad M. Ahmed, M. Megahed

**Affiliations:** 1 Industrial Engineering Program, Department of Mechanical Engineering, College of Engineering, Taif University, P.O. Box 11099, Taif, 21944 Saudi Arabia; 2 Department of Mechanical Engineering, College of Engineering, Taif University, P.O. Box 11099, Taif, 21944 Saudi Arabia; 3 Department of Physics, College of Science, Taif University, Taif, 21944 Saudi Arabia; 4 Department of Mechanical Design and Production Engineering, Faculty of Engineering, Zagazig University, P.O. Box 44519, Zagazig, Egypt

**Keywords:** Engineering, Materials science

## Abstract

Copper and copper alloys are used in industrial applications and food contact surfaces due to their desirable properties; copper metal matrix composites have been exciting researchers' attention in recent years since they can offer many valuable characteristics. The present study investigated the effects of the TiO_2_ nanoparticles addition with different weight percent on the hardness and corrosion behavior of copper nanocomposites. The powder metallurgy method was used to fabricate the Cu/TiO_2_ reinforced with different weight fractions of TiO_2_ nano particles up to 12 wt.%. The corrosion behavior of fabricated specimens is evaluated using potentiodynamic polarization curves and electrochemical impedance spectroscopy in different solutions. These solutions were 3.5wt.% NaCl, 0.5 NaOH and 0.5 M H_2_SO_4_ reflected different pH. The results showed that the addition of TiO_2_ nano particles improves pure copper's hardness. The hardness of pure copper increased from 53 to 91 HV by adding 12 wt.% TiO_2._ The corrosion current density (I_corr_) of copper nanocomposites test specimens was higher than I_corr_ of pure copper in all test solutions. As TiO_2_ nano particles increase, the corrosion resistance of Cu nano composites decreased. All test specimens exhibited little corrosion current density in 3.5 wt.% NaCl solution as compared with other test solutions.

## Introduction

Copper (Cu) and copper alloys are commonly used in industrial applications. Various factors as excellent thermal and electrical conductivities, corrosion resistance, aesthetic appearance, and antimicrobial properties make copper materials are suitable for use in the food sector^1–3^. Cu is commonly used in heating and cooling systems, pipelines for domestic and industrial water utilities containing seawater^4^. On the other hand pure copper suffers from low hardness, low strength under tensile load, and poor wear resistance. For that reason, one of the potential solutions for these weaknesses is the addition of different particles as reinforcement, and produce copper matrix composite^5^. Presently, metal matrix composites are producing a wide range of interest in the future materials, which are the best alternate over the traditional materials^6–9^. At the present time, it is well known that better properties for copper metal matrix composites could be produced by proper reinforcement selection. Different ceramic materials like SiC, Al_2_O_3_, ZrB_2,_ ZrO_2_, TiO_2_ and TiB_2_ have been used as reinforcement particles in the copper matrix. The addition of these reinforcements to copper has led to the improvement of mechanical properties, which have been stated by researchers^10–23^. Elmahdy et al.^5^ reported that, addition 10 wt.% ZrO2 to Cu-, achieved the microhardness (146.5 HV). Zhang et al.^17^ have prepared the ZrB2 reinforced Cu-matrix composites with more than 120 HV hardness. Wang et al.^18^ prepared the ZrB2 reinforced Cu-matrix composites with more than 100 HV hardness. Sreedharan et al.^19^ found that, the hardness of copper increased by increasing B4C nanoparticles addition. Fathy et al.^20^ reported that the hardness of copper increased by increasing Al2O3 nanoparticles addition. Efe et al.^21^ reported that the hardness of copper increased by increasing SiC nanoparticles addition. Although there are many studies that have focused on investigating the effect of nanoparticles on the properties of copper, there are few of them that have been interested in studying Cu matrix composites reinforced with TiO_2_ particles^10,11^. Copper as a metal matrix composites reinforced with TiO_2_ particles are promising materials because of their excellent mechanical and physical properties like good electrical and thermal conductivity and strength at high temperature. Moghanian et al.^10^ studied the effect of addition 1–3 wt.% of TiO_2_ to copper.They found that, the hardness of Cu/TiO_2_ nanocomposite increased by increasing TiO_2_ amount. Sorkhe et al.^23^ the hardness of Cu/TiO_2_ nanocomposite increased by increasing nano particles up to 5 wt.% TiO_2_.

The effect of nanoparticles reinforcements on the corrosion behavior of metal matrix composites is still unclear. Addition of nanoparticles reinforcements may increase or decrease the corrosion resistance of composite materials^23–26^. The corrosion behavior of Cu in aqueous solutions is depending on pH and associating with the morphology of the surface films formed. Few studies have been published about the corrosion behavior of copper composites materials with nanoparticles addition. Saber et al.^24^ found that, in both 3.5wt.%NaCl and 0.5 M H2SO4 solutions, the corrosion rate of Cu/Al_2_O_3_ nanocomposite increased with increasing Al_2_O_3_ content Ghazi et al.^27^ noted that, increasing in SiC as a reinforcement of copper matrix composites, caused severe corrosion at the matrix interface. On the other hand Baghani et al.^28^ stated that, the corrosion current density for the Cu–Zn–Al_2_O_3_ nanocomposite is less than that for the Cu–Zn alloy. It was observed from the investigations made by Hosseini et.al and Rajesh et al.^13,29^ that the corrosion rate of pure copper and copper coated with TiO_2_ were higher compared to Cu/Al_2_O_3_ composites. Ajeel et al.^30^ confirmed that the reinforced copper alloy with 3 wt.% of Al_2_O_3_ and TiO_2_ has a lower corrosion rate than reinforced copper alloy reinforced with 1.5 wt.% of Al_2_O_3_ and TiO_2_.While the reinforced alloys with 1.5 wt.% of Al_2_O_3_ and TiO_2_ has lower corrosion rate compared to the copper alloy. Raghav et al.^31^ studied the corrosion of copper –TiO_2_ nanocomposite coatings on steel. From this study it is concluded that the steel coating with Cu–25TiO_2_ nanocomposite shows better corrosion resistance, than the steel coating with Cu- 20TiO_2_ nanocomposite.

In the present study, the Cu/TiO_2_ reinforced with different weight fractions of TiO_2_ nano particles (0, 4, 8, 12) wt.% were fabricated by powder metallurgy method. The corrosion behavior of fabricated Cu nanocomposites is evaluated. The corrosion behavior is studied using potentiodynamic polarization curves and electrochemical impedance spectroscopy (EIS) in 3.5 wt.% NaCl, 0.5 NaOH and 0.5 M H_2_SO_4_ solutions. In addition, the effect of TiO_2_ nanoparticles on the hardness of Cu nanocomposites is determined. The change in density of copper due to TiO_2_ nanoparticles addition is also determined.

## Experimental work

**Figure 1:**
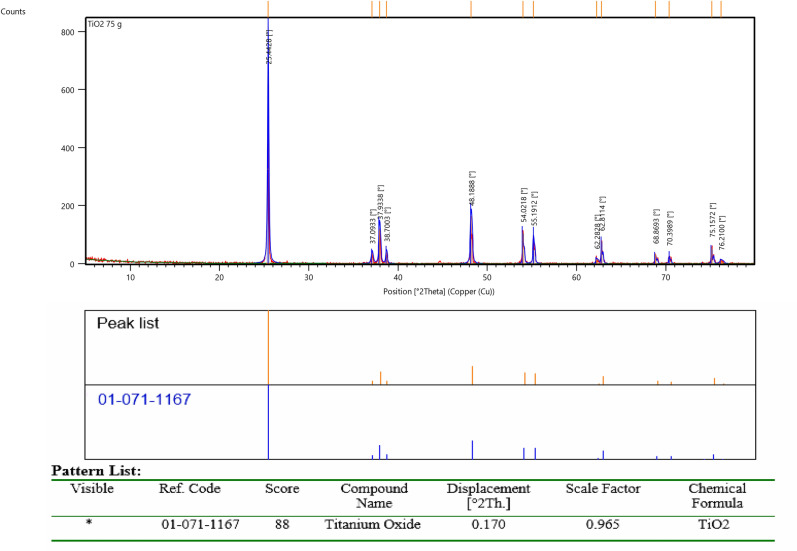
Qualitative XRD analysis of Nano-Titanium Oxide (TiO_2_) used as a reinforcement in Cu-based nanocomposites.

**Figure 2:**
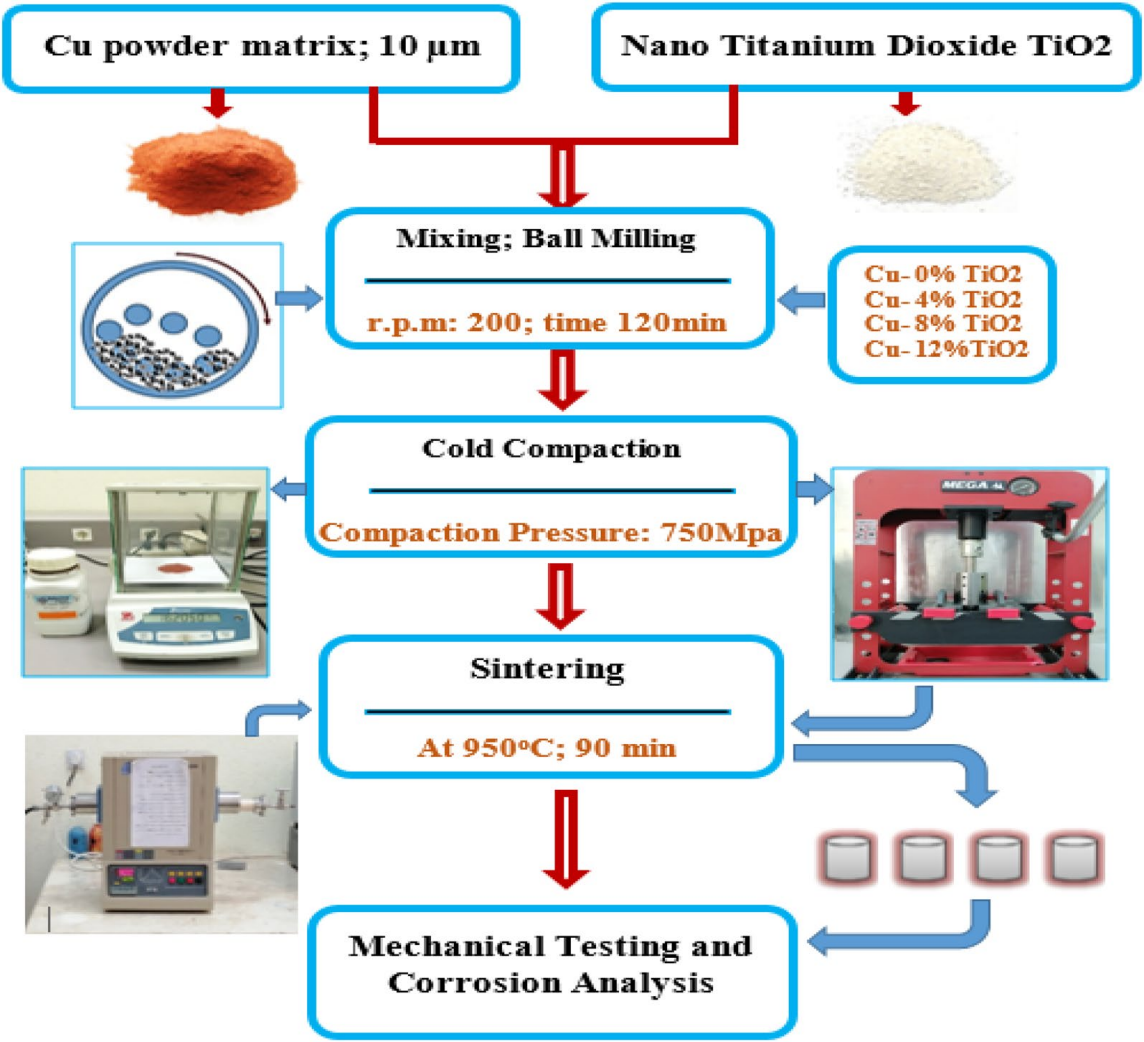
Flow chart and Schematic presentation showing the fabrication path of the present work.

Metal matrix composites (MMCs) containing TiO_2_ nanoparticles with an average particle size of about 80 nm as reinforcements and high purity Cu powder (99% purity and average particle size of 20 μm) as a matrix were prepared by using powder metallurgy method. The chemical analysis of the TiO_2_ nano powder was determined using XRD measurements (Bruker D8 advance diffractometer with a Cu-tube operated at 40 kV and 40 mA). Figure 1 presents the result of qualitative XRD peaks’ profile and phase analysis of the TiO_2_ nano powder used as reinforcement in this study. The metal matrix nanocomposites with weight fractions of 0, 4, 8 and 12 wt.% of TiO_2_ particles were produced. Different weight percentage of Nano TiO_2_ particles was mixed with copper powder using ball mill. Nanocomposite powders were prepared in a way to justify good distribution of the reinforcement particles in the matrix material. For uniform distribution of the reinforcement particles in Cu matrix material, a planetary ball mill (Retsch PM400) for a period of 120 min, with a milling speed of 200 rpm to obtain a uniform distribution of particles was used. The mixed powder of copper and Nano TiO_2_ powder is poured into the cylindrical steel mold with an average internal diameter of 18 mm, external diameter of 60 mm and a height of 80 mm. The powders were compressed at compacted pressure of 700 MPa using hydraulic press with the capacity of 25 ton to prepare the cold compacts from the nanocomposite powders. D2 die steel was used as die material. All specimens after compaction were sintered at 950 °C, for 2 h, in a tube furnace chamber, where the flow of Ar gas was provided^26^. The flowchart of the experimental setup of the Cu/TiO_2_ nanocomposites fabrication path is shown in Fig. 2.

After that, etched with a solution contains 75 ml HCl, 25 ml HNO_3_, 5 ml HF, and 25 ml H_2_O to reveal their microstructure constituents. The microstructure characteristics at the different positions on the specimen surface are investigated by using an optical microscope and scanning electron microscope (SEM). Bulk density measurement of pure Cu and Cu reinforced by TiO_2_ nano particles is obtained by the Archimedes method. On the other hand, the theoretical density is determined using the mixture rule according to the weight fraction of the TiO_2_ nano particles Eq. (1). Porosities of the nancomposites are calculated from difference between the experimental and theoretical density of each sample Eq. (2).

1 $$ \rho_{T} = f_{cu} \cdot \rho_{cu} + f_{p} \cdot \rho_{p} $$

2 $$ porosity = \frac{{\rho_{T} - \rho_{Ex} }}{{\rho_{T} }} \times 100\% $$

where *ρ*, *f* are density and volume fraction or weight fraction. Indices *cu*, *p*, *T* and *Ex* refer to copper, nano particles, theoretical and experimental, respectively^15^.

Microhardness is measured after grinding and polishing processes of the tested specimens using a VHS-1000 microhardnes testing machine at load of 100 g. The corrosion of Cu / TiO_2_ metal matrix composites was accompanied in 3.5 wt.% NaCl, 0.5 M NaOH and 0.5 M H_2_SO_4_ aqueous solutions. Distilled water was used to prepare these solutions prior to each test using. The electrochemical impedance spectroscopy and polarization studies were carried out using Autolab Potentiostat/Galvanostat (PGSTAT 30). The electrochemical impedance spectroscopy measurements were carried out using AC signals of 10 mV amplitude for the frequency spectrum from 100 kHz to 0.01 Hz. A three-electrode cell was used for polarization study. Tafel polarization tests were carried out using a scan rate of 1 mV/min at R.T. The specimens with exposed surface area of 1.7 cm^2^ were used as a working electrode.

## Results and discussion

### Microstructure characteristics

**Figure 3:**
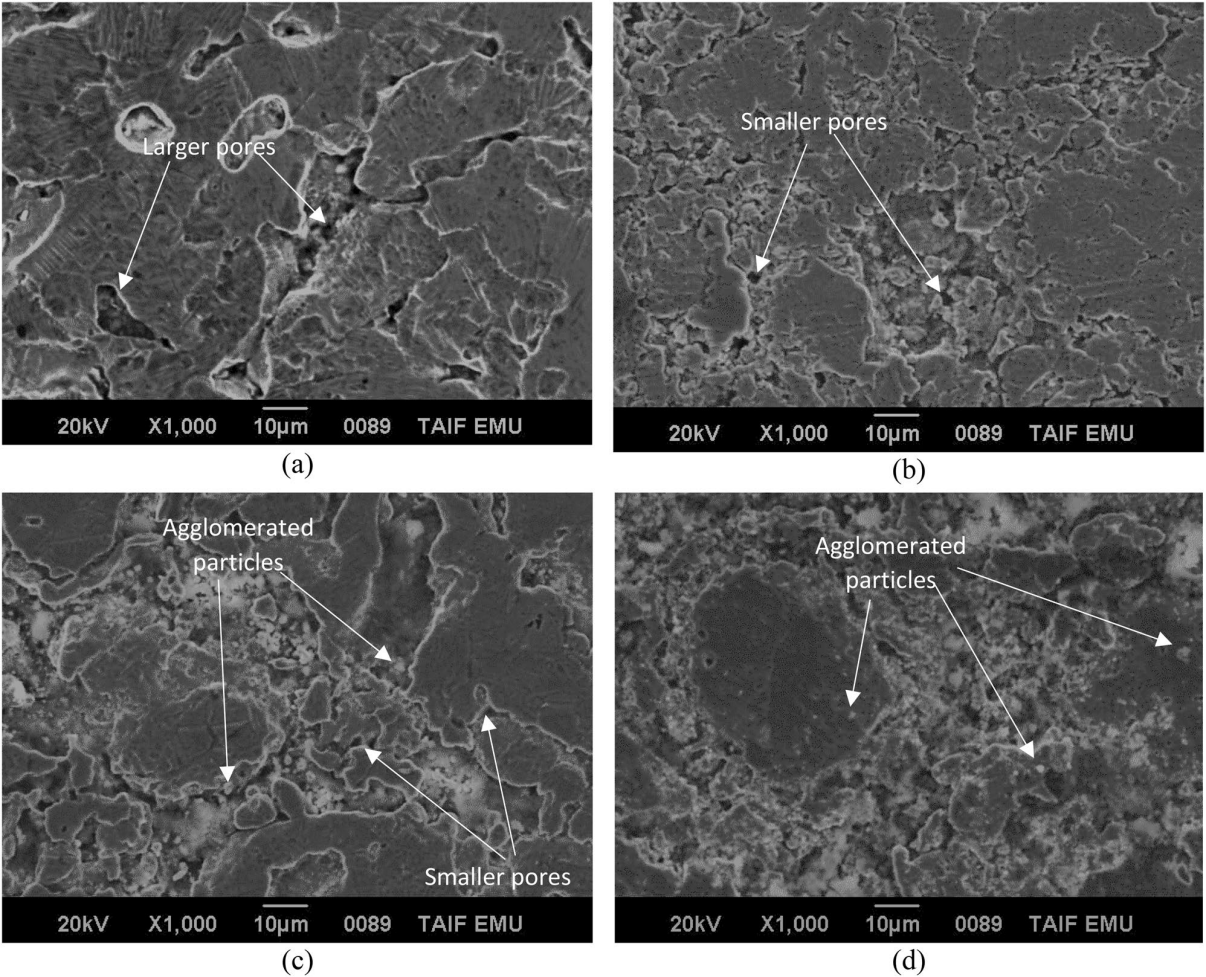
SEM images of the microstructure of Cu/TiO_2_ Nano composites; (**a**) 0% TiO_2_, (**b**) 4% TiO_2_, (**c**) 8% TiO_2_, (**d**) 12% TiO_2_.

**Figure 4:**
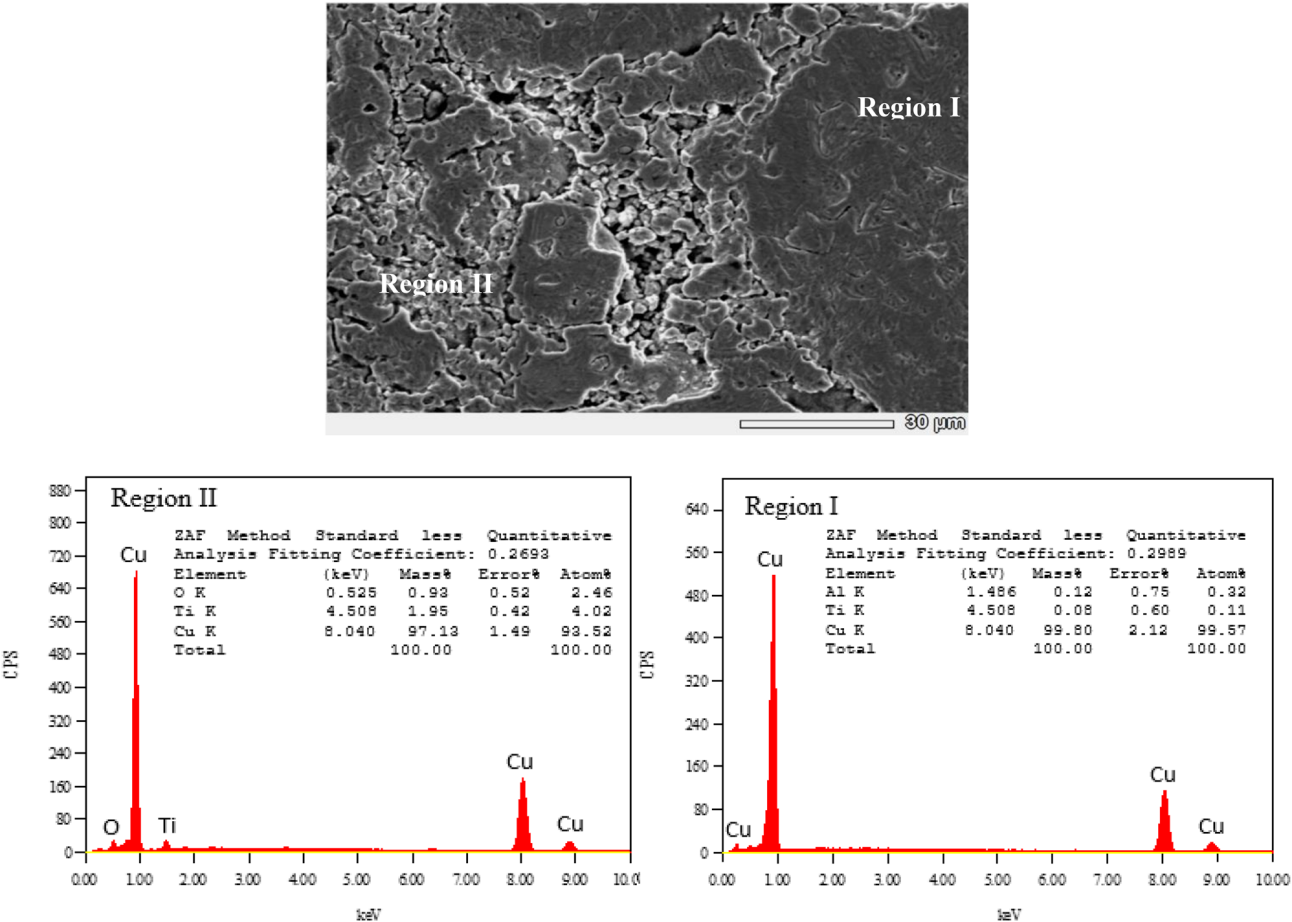
Typical SEM micrograph of different regions and corresponding EDS analysis of Cu/8% TiO_2_ nanocomposite.

**Figure 5:**
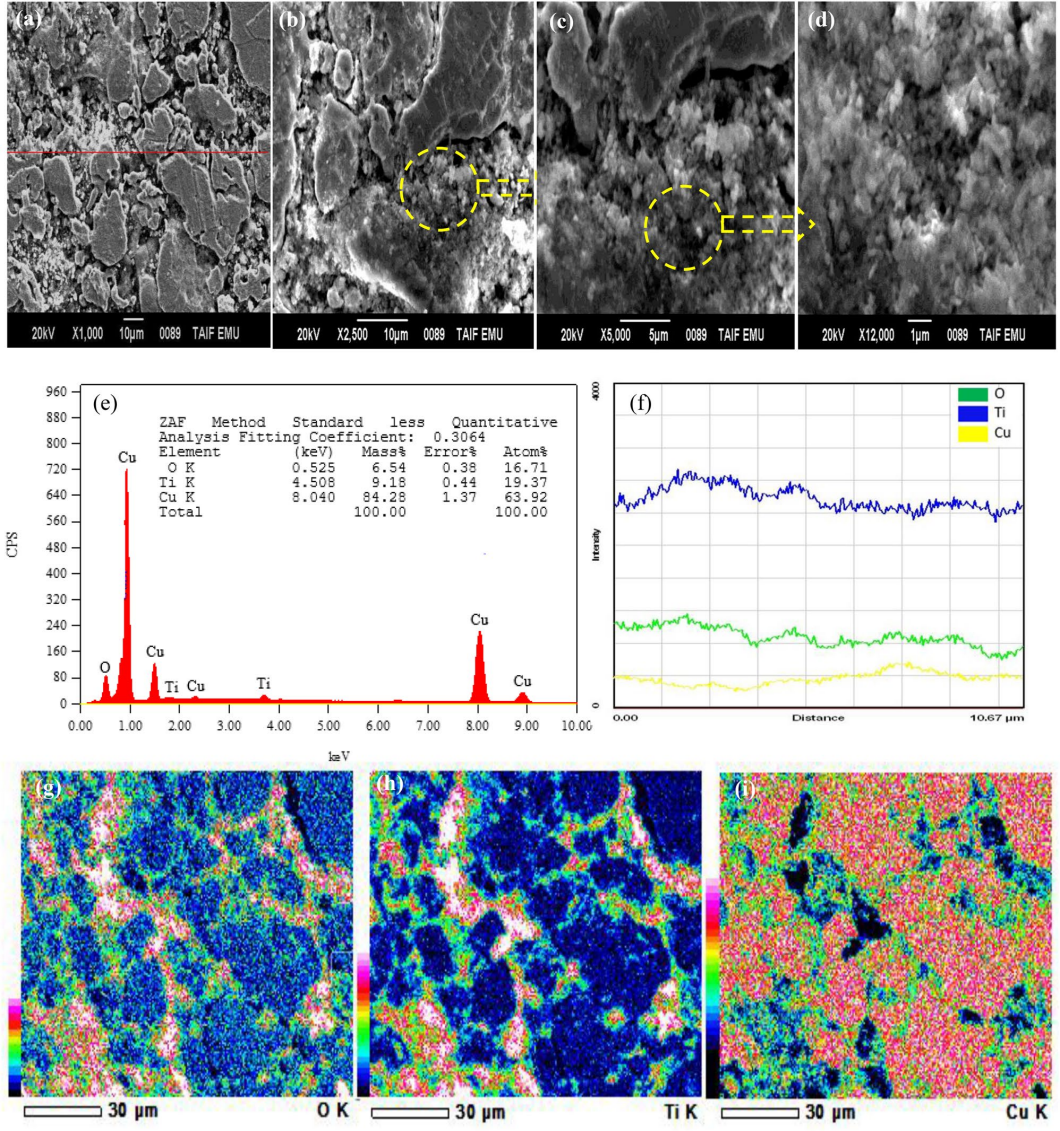
(**a**) SEM image of nanocomposite containing 12 wt.% TiO_2_; (**b**),(**c**),(**d**) Detailed regions of (**a**) with higher magnifications; EDS spectrum analysis of (**a**); (**f**) EDS Line analysis in (**a**); and (**g**),(**h**),(**i**) EDS mappings of Cu, Ti, and O elements present in (**a**).

Figure 3 shows SEM images of the microstructures of synthesized samples of Cu/TiO_2_ nanocomposites with 0%, 4%, 8% and 12 wt.% of TiO_2_. It can be observed the presence of pores in both pure Cu and nanocomposites. As shown in Fig. 3a the amount and size of forming pores in sample without nano TiO_2_ particles, are larger than that in other samples with nano TiO_2_ particles. Also, relatively smaller size of forming pores was observed in the SEM image shown in Fig. 3b, as compared with pure Cu. As shown in Fig. 3c and d smaller amounts and size of pores was observed in the intermediate regions between the Cu matrix structure. This may due to the presence of higher amounts of dispersed nano TiO_2_ particles in these regions and the Cu matrix, but some of these particles agglomerated with increasing in wt.% of TiO_2_ as shown in Fig. 3c,d. This may be attributed that filling capacity of the larder percent of Nano TiO_2_ particles inside Cu matrix^19^. Figure 4 shows SEM microstructure and EDS spectrum analysis of nanocomposite with 8 wt.% of nano TiO_2_ particles. In this figure, SEM micrograph shows the two different regions in the microstructure of the nanocomposite with 8 wt.% of TiO_2_, the first displayed the Cu matrix and the second shows nano TiO_2_ dispersed particles in Cu matrix. EDS spectrum analysis of nanocomposite with 8 wt.% of nano TiO_2_ particles and corresponding elements composition are given in Fig. 4. This confirms the existence of nano TiO_2_ particles in Cu matrix structure. Typical higher magnification SEM micrographs and corresponding EDS analysis of Cu/12% TiO_2_ nanocomposite with line analysis and EDS mapping are displayed in Fig. 5a–i. As shown in this figure, the results of surface scanning obtained by line analysis and elemental EDS mapping of Cu, Ti, and O elements present in nanocomposites show a uniform distribution of TiO_2_ particles in the structure of nanocomposite. But, some of these particles were agglomerated with increasing in wt.% of TiO_2_ In this figure it is obvious that copper covers almost the entire surface of the microstructure. The results of surface scanning for Ti and oxygen show that these two elements are present less in the microstructure of the nanocomposite material and the surfaces they inhabit are inter-lapping, which corresponds to the existence of TiO_2_ dispersion in the microstructure. In addition, these figure reveals the presence of larger amount of second dispersed phase particles, and the homogeneous dispersion of TiO_2_ in the Cu matrix for the nanocomposite specimens.

### Density and porosity measurements

**Figure 6:**
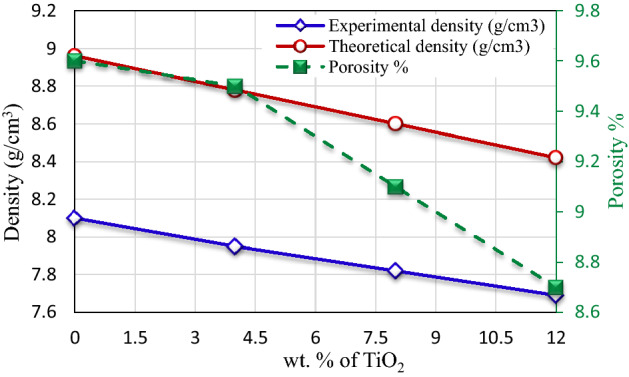
Correlation between both the density and porosity with nano-TiO_2_ content.

Figure 6 presented the correlation between the density and porosity with the different wt.% of TiO_2_ Nano-particles. From the figure, it is clear that the theoretical densities of nanocomposites decreased linearly, as expected for the mixtures rule. In addition, the TiO_2_ density was lower than the pure Cu. Therefore, any increasing in TiO_2_ content must decrease the density of the nanocomposite. The experimental densities are lower than the theoretical densities of all test specimens. This is because the fabricated nanocomposites may contain some porosity. According to Fig. 6 the porosity in nanocomposites decreased gradually with increasing in weight fraction of the TiO2 Nano-particles. As shown in Table 1 there is no great difference in the porosity between copper and copper nanocomposites. The porosity in copper was 9.6%. while it recorded 9.5% in nanocomposite with 4 wt.% TiO_2._ On the other hand a little decrease was noted in nanocomposite with 12 wt.% TiO_2_ and recorded about 8.7%. This result was in agreement with Norouzifard et al.^15^ and Saif et al.^32^. Norouzifard et al.^15^ fabricated Cu metal matrix composites contain 2.5, 5.5, and 8 wt.% steel nanoparticles. They found that porosity reduces by increasing the steel particles weight fraction. Saif et al.^32^ using the powder metallurgy technique to fabricate Al/TiO_2_ nanocomposite with different content of nano-TiO2 particles. They found that, by increasing wt% of TiO_2_ nanoparticles in the composite matrix the porosity decreases gradually. This can be attributed to diffusion enhancement with increase of sintering time, which causes disappearance of voids between powder particles^33^. Moreover, the nanoparticles possess high penetration ability within the pores and voids of the nanocomposite matrix^32^. Malek et al.^34^ observed that the number of pores decreased at high sintering temperatures. At the high sintering temperature, the matrix was moved to fill the voids during the consolidation. Kamrani et al.^35^ suggested that the diffusion of the matrix into the interparticle pores is responsible for this observation.

**Table 1:** Physical and mechanical properties of Cu/TiO_2_ nanocomposites samples.

Nano TiO_2_-content (wt.%)	Density (g/cm^3^) experimental	Density (g/cm^3^) theoretical	Porosity%	Microhardness (Hv)
0	8.1	8.96	9.6	53
4	7.95	8.78	9.5	68
8	7.82	8.6	9.1	79
12	7. 69	8.42	8.7	91

### Hardness

Microhardness results of the test specimens are shown in Table 1. As recorded in the table, the microhardness increases with increasing TiO_2_ Nano_-_particles. The hardness of pure Cu was 53 HV, and increased to 91 HV, in Cu nanocomposite with 12 wt% TiO_2_. The addition of 4 wt.% TiO_2_ Nano_-_particles improves pure Cu's hardness by 28.3%. Furthermore, by adding of 12wt.% TiO_2_ Nano_-_particles improve the hardness of pure Cu by 71.7%. This improvement in the hardness of Cu/TiO2 nanocomposites is due to the hardness of pure TiO_2_ Nano_-_particles was higher than pure Cu. Zawrah et al.^14^ concluded that the addition of Al_2_O_3_ nano particles to pure Cu improved the hardness of nanocomposites materials. This because of the existence of Al_2_O_3_ nano particles and their uniform distribution as a strength-enhancing agent. Ning et al.^11^ prepare Cu/TiO_2_ nanocomposite coatings with different content of TiO_2_ nano particles. The nanocomposite coating Cu/25 wt.% TiO_2_ presented considerably improved microhardness of 218.7 Hv. They suggests that small grain size of nanoparticles has a very strong effect on hardening of Cu/%TiO_2_ nanocomposite. The Orowan mechanism plays a remarkable role on the strengthening of the composites, particularly when the reinforcement size is less than 100 nm^23^. The Orowan mechanism proposes that the existence of non-shearable TiO2 particles within the matrix causes dislocation loop to be left behind after a dislocation line has passed through ceramic TiO_2_ particles. It also hinders and/or slows down dislocation motion in copper metal matrix. The internal strain created during the milling process and TiO_2_ nanoparticles distributed in a copper matrix act as dislocation movements barrier are the other reasons for hardness increase. Vishwanath et al.^37^ also explained the reasons for increasing copper's microhardness related to ceramic nanoparticles' addition. They suggested that, by adding of strong and stiff ceramic nanoparticles in the soft ductile copper led to enhancement in the microhardness of copper metal matrix nanocomposites. Another reason is the difference in coefficient of expansion between copper matrix and ceramic nanoparticles can lead to formation of dislocations. The increase in ceramic nanoparticle content led to nanocomposites' dislocation density and acted as obstacles for plastic deformation.

### Electrochemical measurements

**Figure 7:**
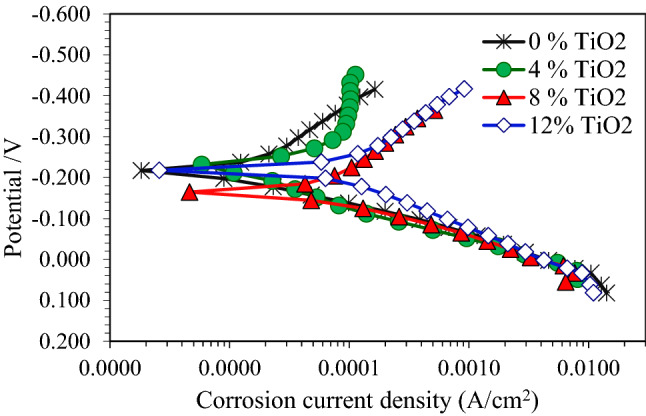
Polarization curves for copper metal matrix composites in 3.5wt.% NaCl solution.

**Figure 8:**
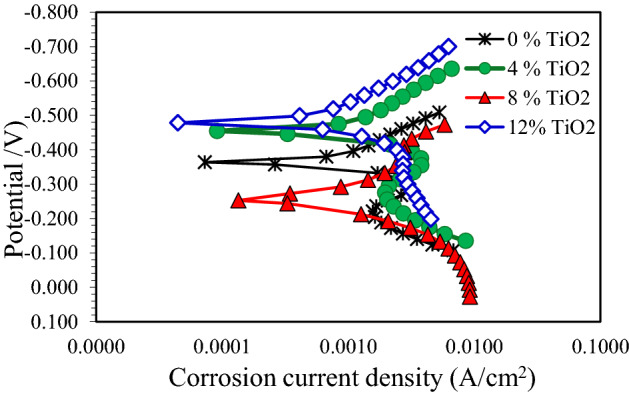
Polarization curves for copper metal matrix composites in 0.5 M NaOH solution.

**Figure 9:**
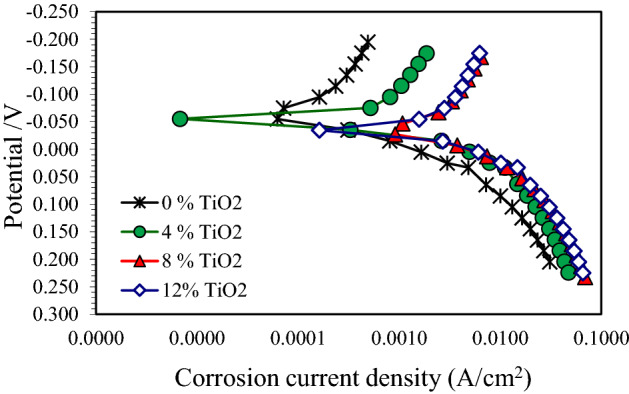
Polarization curves for copper metal matrix composites in 0.5 M H_2_SO_4_ solution.

The polarization curves of the pure Cu and nanocomposite samples with 4, 8 and 12 wt.% TiO_2_ after corrosion tests in 3.5% NaCl, 0.5 M NaOH and 0.5 M H_2_SO_4_ solutions are indicated in Figs. 7, 8 and 9. These figures showed that the addition of TiO_2_ in pure Cu matrix increased the anodic and cathodic current densities, and resulted in significant increase in the corrosion current density in all test solutions. Corrosion potential (E_corr_) and corrosion current density (I_corr_) were determined by Tafel extrapolation method and they displayed in Tables 2, 3 and 4.The polarization curves Fig. 7 for pure Cu and its nanocomposite specimens in 3.5% NaCl solution shows that active dissolution in anodic region. Also it can be observed that, there is no great difference in E_corr_ between Cu nanocomposite specimens and the pure Cu. As shown in Table 2, I_corr_ of pure Cu in 3.5% NaCl solution was 0.01 mA/cm^2^ and it raised to 0.026 mA/cm^2^ with 4 wt.% TiO_2_ nano particles. The severity of corrosive attack continuous increased with the addition of TiO_2_ nano particles, and recorded I_corr_ 0.063 mA/cm^2^ for nanocomposite with 12 wt.% TiO_2_. The anodic reactions in NaCl solution may be as following reactions^36–39^, at first, the oxidation of copper transforms the copper to Cu + ion (Eq. 3). In the presence of aggressive chloride ions, the reaction between Cl^−^ and Cu + occurs, producing a soluble film on the surface (Eq. 4).

3 $$ {\text{Cu}} \to {\text{Cu}}^{ + } + {\text{e}}^{ - } $$

4 $$ {\text{Cu}}^{ + } + {\text{Cl}}^{ - } \to {\text{CuCl}} $$

**Table 2:** Corrosion properties of Cu/TiO_2_ with different TiO_2_ content in 3.5% NaCl solution.

Nano TiO_2_ content (wt.%)	Electrochemical parameters		Impedance measurements	
	Corrosion current density. (mA/cm^2^)	Corrosion potential E_corr_.(− mV)	Ru (ohm)	Rp (ohm)
0	0.01	217	35.33	13.99
4	0.026	215	33.57	9.48
8	0.048	165	16.7	6.53
12	0.063	218	13.8	5.2

**Table 3:** Corrosion properties of Cu/TiO_2_ with different TiO_2_ content in 0.5 M NaOH solution.

Nano TiO_2_ content (wt.%)	Electrochemical parameters		Impedance measurements	
	Corrosion current density. (mA/cm^2^)	Corrosion potential E_corr_.(− mV)	Ru (ohm)	Rp(ohm)
0	0.67	364	10.67	7.01
4	1.26	455	10.58	5.7
8	1.43	253	7.7	3.64
12	1.93	479	5.2	2.9

**Table 4:** Corrosion properties of Cu/ TiO_2_ with different TiO_2_ content in 0.5 M H_2_SO_4_ solution.

Nano TiO_2_ content (wt.%)	Electrochemical parameters		Impedance measurements	
	Corrosion current density (mA/cm^2^)	Corrosion potential E_corr_.(− mV)	Ru (ohm)	Rp (ohm)
0	1.2	35.1	5.2	4.9
4	2.7	53.3	4.5	4.6
8	3.6	14.5	2.1	2.9
12	5.3	34.5	1.03	1.87

CuCl is an unstable film and instantly reacts with Cl ions and changes to CuCl^−2^ as shown in Eq. (5):

5 $$ {\text{CuCl}} + {\text{Cl}}^{ - } \to {\text{CuCl}}^{{ - {2}}} $$

The anodic dissolution of copper was organized by both electro dissolution of copper and diffusion of soluble CuCl^−2^ to NaCl solution^39^. As CuCl^−2^ formed by anodic dissolution low protection surface is expected^38^.

Figure 8 shows the polarization curves of pure Cu and its nanocomposite specimens in 0.5 M NaOH solution. This figure indicated a short passive area in the anodic region, specifically for pure Cu and nanocomposite with 4% TiO_2_. In addition, the corrosion potential shifts towards more active potentials as TiO_2_ nano particles was 12%. From Table 3, E_corr_ of pure Cu in 0.5 M NaOH solution was − 364 mV, where it was − 479 mV for nanocomposite with 12 wt.% TiO_2_. I_corr_ of pure Cu in 0.5 M NaOH solution was 0.67 mA/cm^2^ and it raised to the highest value of 1.93 mA/cm^2^ with 12 wt.% TiO_2_ nano particles.

The anodic behavior of copper in NaOH solution may form an oxide film consisting of either Cu_2_O or a duplex layer of Cu_2_O and CuO depending on the electrolyte composition and electrochemical conditions^40^. In NaOH solution below 1 M NaOH concentration the direct dissolution of copper as cuprite ions according to either (or both) of the following reactions (Eqs. 6,7):

6 $$ {\text{Cu}} + {\text{3OH}}^{ - } \to {\text{HCuO}}_{{2}}^{ - } + {\text{H}}_{{2}} {\text{O}} + {\text{2e}}^{ - } $$

7 $$ {\text{Cu}} + {\text{4OH}}^{ - } \to {\text{Cu}}\left( {{\text{OH}}} \right)_{{4}}^{{{2} - }} + {\text{2e}}^{ - } $$

and/ or from Cu(OH)_2_ through chemical reaction (Eq. 8):

8 $$ {\text{Cu}}\left( {{\text{OH}}} \right)_{{2}} + {\text{2OH}}^{ - } \to {\text{CuO2}}^{{{2} - }} + {\text{2H}}_{{2}} {\text{O}} $$

In noble potential the oxidation of cuprous oxide to either (or both) CuO and Cu (OH)_2_ according to the following equations:

9 $$ {\text{Cu}}_{{2}} {\text{O}} + {\text{2OH}}^{ - } \to {\text{2CuO}} + {\text{H}}_{{2}} {\text{O}} + {\text{2e}}^{ - } $$

10 $$ {\text{Cu}}_{{2}} {\text{O}} + {\text{2OH}}^{ - } + {\text{H}}_{{2}} {\text{O}} \to {\text{2Cu}}\left( {{\text{OH}}} \right)_{{2}} + {\text{2e}}^{ - } $$

The resulting Cu (OH)_2_ may be found in the following equilibrium.

11 $$ {\text{Cu}}\left( {{\text{OH}}} \right)_{{2}} \to {\text{CuO}} + {\text{H}}_{{2}} {\text{O}} $$

At pH (> 13), thermodynamic equilibrium consideration shows that CuO is unstable and likely dissolves as HCuO_2_^−^/CuO2^2−^ ^41^.

In Fig. 9, it is obvious that the nanocomposite specimens with 4 wt.% , 8 wt.% and 12 wt.% TiO_2_ tested in 0.5 M H_2_SO_4_ solution revealed approximately the same behavior in both anodic and cathodic regions as well as pure Cu. The corrosion reaction of Cu in H_2_SO_4_ is as follows:

12 $$ {\text{Cu}} + {\text{4H}}^{ + } + {\text{SO}}_{{4}}^{{ - {2} }} \to {\text{Cu}}^{{ + {2}}} + {\text{SO}}_{{2}} + {\text{H}}_{{2}} {\text{O}} $$

**Figure 10:**
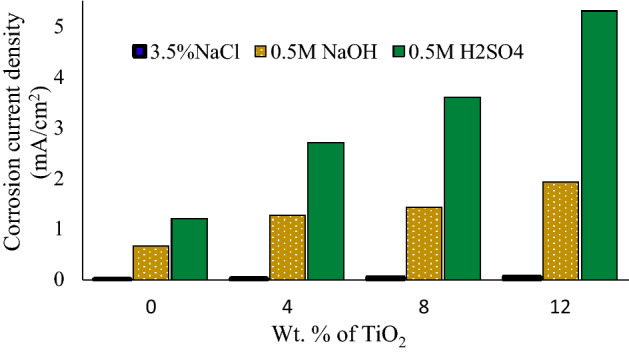
Corrosion current density (i_corr_) for the three Cu/TiO_2_ nanocomposites in different corrosive media (3.5% NaCl, 0.5 M NaOH, and 0.5 M H_2_SO_4_).

The presence of SO_4_^–2^ ions on the surface increases the attack on the copper surface^42^. By comparing the results of corrosion current densities of the investigated nanocomposites and pure Cu in different test solutions, it can be seen that I_corr_ of all specimens is elevated in 0.5 M H_2_SO_4_ solution in comparison with 3.5 wt.% NaCl and 0.5 M NaOH solutions according to Fig. 10.

**Figure 11:**
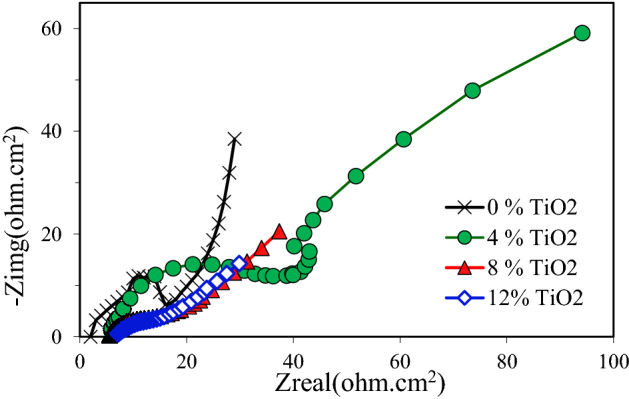
Nyquist plots of copper metal matrix composites in 3.5wt.% NaCl Solution.

**Figure 12:**
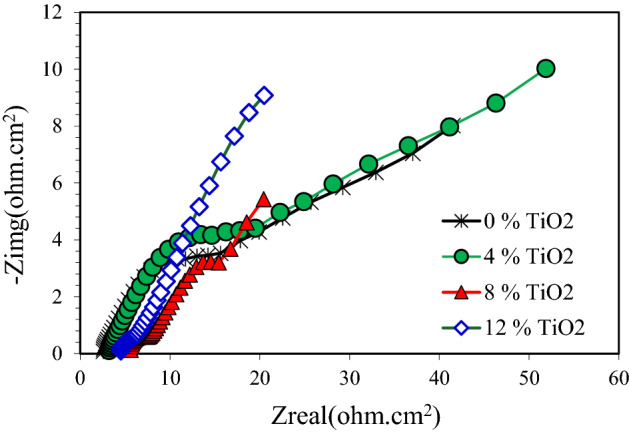
Nyquist plots of copper metal matrix composites in in 0.5 M NaOH Solution.

**Figure 13:**
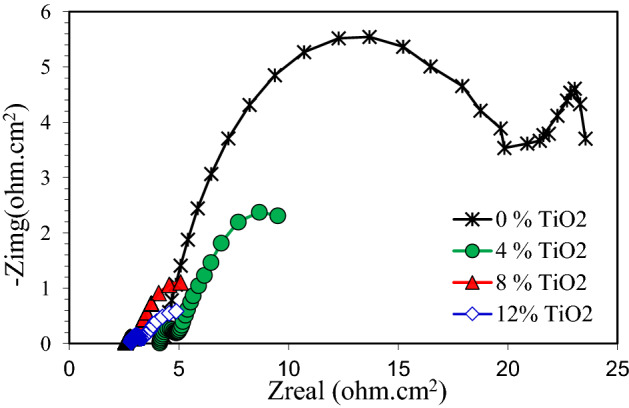
Nyquist plots of copper metal matrix composites in 0.5 M H_2_SO_4_ Solution.

The electrochemical impedance spectroscopy is a powerful means that can be used to disentangle the mechanism of electrochemical reactions^43,44^. Figure 11 shows the Nyquist diagrams for pure Cu and nanocomposites exposed to 3.5 wt% NaCl solution. An arc was observed for pure Cu and nanocomposite with 4 wt.% TiO2, followed by a second arc or tail. From the same figure, it was visible that the nanocomposite Nyquist curve begins approximately at 7 Ohm-cm^2^. This indicates that the migration of the corrosion products by ions to the solution was possible. As a result, this concluded that the nanocomposite a weak and leaky layer that can be dissolved over time permitting the continuous corrosion of the nanocomposite. Figure 12 shows the Nyquist diagrams for pure Cu and Cu nanocomposites exposed to 0.5 M NaOH solution. This figure shows that the Nyquist diagram for pure Cu displays a small semicircle followed by a straight line. Nyquist diagram for Cu nanocomposite with 4% TiO_2_ shows a smaller semicircle than pure Cu followed by a straight line. It is obvious from Fig. 13 that, the obtained Nyquist diagram of pure Cu in H_2_SO_4_ solution produce a semi-circular shape. This indicates that charge transfer essentially controls the corrosion process. The measured electrochemical impedance spectroscopy results for the Cu and Cu nanocomposites in 3.5% NaCl, 0.5 M NaOH and 0.5 H_2_SO_4_ solutions are summarized in Tables 2, 3 and 4. According to results recorded in Tables 2, 3 and 4, addition of TiO_2_ Nano particles to Cu matrix decreased the resistances Rp and Ru. Another observation, the resistance values Rp and Ru of the specimens in the 3.5% NaCl solution are larger than those in the 0.5 M NaOH and 0.5 H_2_SO_4_ solutions. Furthermore, the specimens' resistance values Rp and Ru in 0.5 H_2_SO_4_ solution are the smallest of the three solutions as shown in Tables 2, 3 and 4. The obtained results from polarization curves agree with electrochemical impedance spectroscopy results.

**Figure 14:**
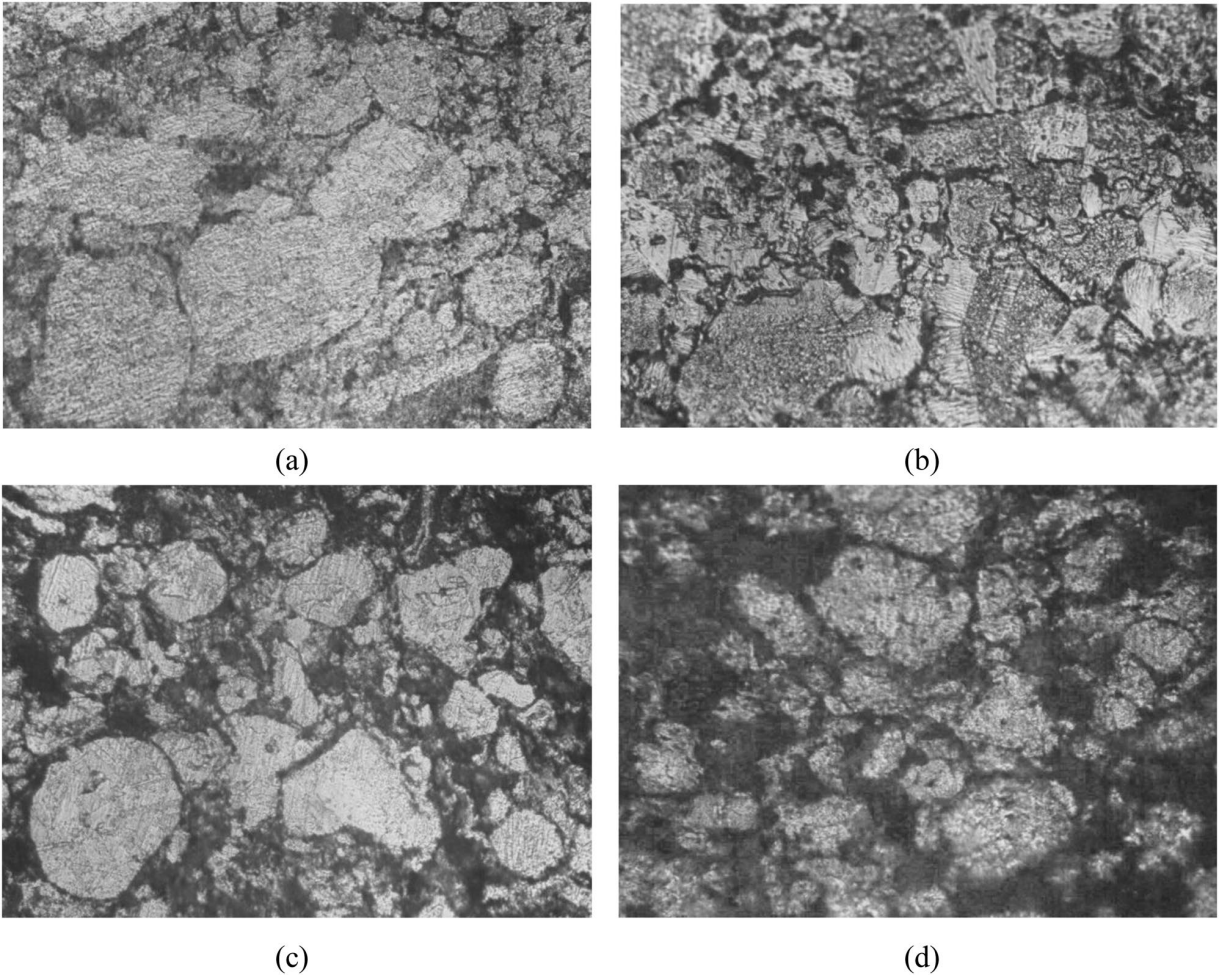
Corroded surfaces of C/TiO_2_ nanocomposites, in 3.5% NaCl solution, X200; (**a**) 0% TiO_2_, (**b**) 4% TiO_2_, (**c**) 8% TiO_2_, (**d**) 12% TiO_2_.

The microstructure of corroded surface of pure Cu and Cu nanocomposite after corrosion test in 3.5% Nacl solution is shown in Fig. 14(a–d). From the figure, it is clear that pure Cu and Cu nanocomposite were attacked by pitting corrosion. In Fig. 14a, pure Cu shows fine pits distributed over the structure, while this pits increased after 4wt.% TiO_2_ Nano-particles addition as shown in Fig. 14b. Additionally, these pits increased with increasing TiO_2_ Nano-particles addition as shown in Fig. 14a–d. Another observation, Cu-based nanocomposites show that the attacked areas were mainly concentrated around TiO_2_ Nano-particles. Moreover, the test solution had slight etching effect on these specimens.

The present results of polarization curves and electrochemical impedance spectroscopy measurements of corrosion behavior of pure Cu and its nanocomposites showed that, the addition of TiO_2_ nano-particles to Cu increase corrosion current denesity in different test solutions. One possible reason for this is that Cu nanocomposites specimens may have a higher initial susceptibility to corrode compared to pure Cu due to the attendance of TiO_2_ nano-particles. Harovel^44^ reported that the composite materials may corrode in the interfacial area due to the residual stresses between the particles and the matrix material. Arsenault^45^ reported that, microstructural features may effect the composite materials because of the presence of the reinforcements, and intermetallic phases may be formed around reinforcements. In addition, dissimilarities in the coefficient of thermal expansion between ceramic reinforcement and metal matrix can lead to the generation of dislocations during heating and cooling of metal matrix composite. These dislocations may lead to higher corrosion in metal matrix composite.

The metal matrix and reinforcement exhibit different electrochemical corrosion potentials and characteristics in a neutral salt spray environment^46,47^.The addition of reinforcement to matrix alloy changes the homogeneity of structure making the matrix more susceptible to localized corrosion^48^. In addition the difference of the reinforcement phase, led to different corrosion behavior of composite materials^24,27^. Ghazi et al.^28^ noted that increasing SiC as a reinforcement of copper matrix composites caused severe corrosion at the matrix interface. Saber et al.^25^ found that, in both 3.5 wt.% NaCl and 0.5 M H_2_SO_4_ solutions, the corrosion rate of Cu/Al2O3 nanocomposite increased with increasing Al2O3 content. Rajesh et al. and Hosseini et al. ^13,29^ found that the corrosion rate of pure copper and copper coated with TiO2 was higher than Cu/Al_2_O_3_ composites. Naseri et al.^23^ recommended that, in Cu/TiO2interface, the galvanic couple existes and Cu acts as the anode while it acts as the cathode. It can be concluded that the potential difference between Cu and TiO_2_, corrosion process of Cu is accelerating, especially the corrosion rate of the area adjacent to TiO2particles. It can be predicted that corrosion of TiO_2_ particles is extremely slight, and its main corrosion type could be pitting. Usually, cuprous oxide is formed when copper and oxygen react in presence of chloride ions. But increase in ceramic particles increases the rate of corrosion as the oxidation reaction speeds up due to the presence of more oxide particles in the layer and formation of thick unstable copper peroxide layer. Since the time for passivating layer (Cuprous oxide) to form is greater than the time for oxide layer (Copper peroxide) to form, degradation was more on the top layer of the composite which was also observed in some previous works^13,24,27^. This could also be seen in the decreasing polarization resistance value. It may be inferred that the increasing in ceramic particles content the copper metal matrix composite was more susceptible to corrosion and becomes unsuitable for use in corrosive environments^13^.

## Conclusions

Copper metal matrix nanocomposite has desirable properties for various applications such as heating and cooling systems, pipelines and drinking vessels. Cu-based nanocomposites with different wt.% of TiO_2_ were fabricated and their properties were evaluated. The Cu density was decreased due to add TiO_2_ nanoparticles. In addition, the experimental densities of fabricated specimens were lower than the theoretical densities of all test specimens. This is because the fabricated nanocomposites may contain some porosity. The hardness of pure Cu was 53 HV, and increased to 91 HV, in Cu-based nanocomposite with 12 wt.% TiO_2_ with improving ratio 71.7%. The electrochemical measurements of pure Cu and Cu reinforced with 0, 4, 8 and 12wt% TiO_2_ nano particles was studied in 3.5% NaCl, 0.5 M NaOH and 0.5 M H_2_SO_4_ solutions by Potentiodynamic polarization curves and electrochemical impedance spectroscopy (EIS). The corrosion current density of pure Cu increases with the increasing of TiO_2_ nanoparticles percentage in all tested solutions. In addition, the corrosion current density of all test specimens in 0.5 M H_2_SO_4_ solution was higher than the corrosion current density of test specimens in both 0.5 M NaOH and 3.5% NaCl solutions. This is because the acidic solution is more severe than both the alkaline and salty solutions. The results of the measured impedance for the pure Cu and Cu matrix composites in 3.5% NaCl, 0.5 M NaOH, and 0.5 H_2_SO_4_ solutions confirm the results obtained from the potentiodynamic polarization curve. Furthermore, the specimens' resistance values Rp and Ru in the 3.5% NaCl solution are larger than those in the 0.5 M NaOH and 0.5 H_2_SO_4_ solutions. It can be concluded that Cu matrix composites reinforced with TiO_2_ particles may be promising materials due to their excellent mechanical and physical properties. However the corrosion behavior need more studies.
